# Incremental Value of Iodine-125 Seed Implantation After Bronchial Artery Chemoembolization in Immunotherapy-Treated Advanced Lung Squamous Cell Carcinoma with Hemoptysis: A Retrospective Cohort Study Using Inverse Probability of Treatment Weighting

**DOI:** 10.3390/curroncol33070402

**Published:** 2026-07-05

**Authors:** Linhao Ran, Jiangwei Chen, Huan Liang, Jiajian Xie, Weichen Fu, Dichun Yang, Fan Li, Ying Liu, Li Jiang

**Affiliations:** 1 Department of Nuclear Medicine, Banan Hospital of Chongqing Medical University, Chongqing 401320, China; 137143@hospital.cqmu.edu.cn (L.R.);; 2 Department of Ultrasound, Banan Hospital of Chongqing Medical University, Chongqing 401320, China

**Keywords:** hemoptysis, lung squamous cell carcinoma, immunotherapy, BACE, Iodine-125 seed

## Abstract

Background: The incremental value of iodine-125 (I-125) seed implantation in advanced refractory lung squamous cell carcinoma (LUSC) with hemoptysis treated with bronchial artery chemoembolization (BACE) and immunotherapy remains unclear. Methods: This retrospective cohort study included 90 patients treated between June 2023 and June 2025. Patients receiving BACE plus immunotherapy were classified according to whether I-125 seed implantation was performed within 7 days after BACE: G1, BACE plus immunotherapy (*n* = 42), and G2, BACE, I-125 seed implantation, and immunotherapy (*n* = 48). Inverse probability of treatment weighting (IPTW) served as the primary adjustment method. Results: After IPTW, baseline covariates were well balanced; propensity score matching yielded 26 patients per group. Compared with G1, G2 was associated with longer hemoptysis-free survival (not reached vs. 11 months; HR = 0.34, 95% CI 0.18–0.64, *p* < 0.05), overall survival (19 vs. 14 months; HR = 0.26, 95% CI 0.15–0.44, *p* < 0.05), and progression-free survival (12 vs. 9 months; HR = 0.34, 95% CI 0.21–0.56, *p* < 0.05). The 6-month objective response rate (ORR) and disease control rate (DCR) were higher in G2, whereas no significant difference in 24-h hemostasis or recorded grade 3 or higher adverse events was observed. Conclusions: Adding I-125 seed implantation to BACE plus immunotherapy was associated with improved outcomes in selected patients, and prospective validation is warranted.

## Simple Summary

Hemoptysis is a serious and potentially life-threatening problem in patients with advanced lung squamous cell carcinoma. Catheter-based treatment can rapidly control bleeding by blocking tumor-feeding arteries and delivering local chemotherapy, but recurrent bleeding and tumor progression remain common challenges. This study evaluated whether the addition of iodine-125 seed implantation, a form of internal radiotherapy, could provide further benefit for patients receiving catheter-based treatment and immunotherapy. In this retrospective cohort, patients who underwent iodine-125 seed implantation after catheter-based treatment had longer bleeding-free survival, better tumor response, and longer overall survival compared to those treated without seed implantation. These findings suggest that iodine-125 seed implantation may serve as a useful local treatment option in carefully selected patients. Future prospective studies are needed to confirm these results and to establish standardized treatment and safety protocols.

## 1. Introduction

Lung cancer remains one of the leading causes of cancer incidence and cancer-related mortality worldwide. Lung squamous cell carcinoma (LUSC) is a major histological subtype of non-small cell lung cancer and is strongly associated with tobacco exposure, occupational carcinogens, and air pollution. Its development is generally linked to squamous metaplasia, dysplasia, and progression to invasive carcinoma. Standard therapeutic modalities for LUSC include surgery, radiotherapy, chemotherapy, and local palliative interventions. However, advanced refractory lung squamous cell carcinoma (LUSC) remains difficult to manage after disease progression or intolerance following radiotherapy and chemotherapy [1]. Immune checkpoint inhibitors (ICIs) have improved systemic treatment options for this population, but responses remain heterogeneous and local tumor progression can still precipitate serious complications [2]. Tumor-related hemoptysis is particularly dangerous because massive bleeding may obstruct the airway and can be fatal [3,4]. Recurrent bleeding can also interrupt systemic therapy and reduce the clinical benefit expected from immunotherapy.

Bronchial artery chemoembolization (BACE) serves as a primary treatment for tumor-related hemoptysis [5]. This procedure achieves high short-term hemostasis rates by embolizing bleeding vessels while delivering local chemotherapy, thereby balancing immediate hemostasis with antitumor activity [6,7]. However, despite its initial success, persistent tumor growth, collateral circulation formation, and vascular recanalization confer a substantial medium- to long-term risk of rebleeding [8,9]. These limitations underscore the challenge of achieving durable local control with conventional interventional therapy.

These limitations leave a need for local treatment that can act in parallel with systemic therapy and maintain tumor control long enough to lower the risk of recurrent hemoptysis [10]. I-125 seed brachytherapy is one candidate approach. Implanted seeds release low-dose gamma radiation over time, causing DNA damage and sustained irradiation within the tumor [11,12,13]. This technique has been used for local control in solid tumors, including prostate and esophageal cancers [14,15,16]. On this basis, we examined whether adding I-125 seeds to BACE could link rapid hemostasis with a continued local radiation effect. The rationale is to reduce residual tumor burden and invasion of nearby vessels or tissues while maintaining compatibility with immunotherapy as part of a local-systemic treatment plan.

Our previous exploratory institutional study suggested that BACE combined with I-125 seed implantation may be feasible and clinically active in patients with advanced LUSC-associated hemoptysis [17]. However, that study was not designed to evaluate the incremental value of I-125 seed implantation in patients receiving immunotherapy, nor did it use BACE plus immunotherapy as a comparator. Therefore, whether adding I-125 seed implantation to BACE and immunotherapy provides additional benefit remains uncertain.

Thus, this retrospective cohort study aimed to compare clinical outcomes between BACE plus immunotherapy and BACE followed by I-125 seed implantation plus immunotherapy in patients with advanced refractory LUSC complicated by hemoptysis. The primary objective was to evaluate whether I-125 seed implantation was associated with more durable hemoptysis control and improved survival outcomes.

## 2. Materials and Methods

### 2.1. General Information

This retrospective cohort study included 90 patients with advanced refractory lung squamous cell carcinoma complicated by hemoptysis who were treated at Banan Hospital of Chongqing Medical University between June 2023 and June 2025. The study was approved by the Ethics Committee of Banan Hospital of Chongqing Medical University (approval number: BNLL-KY-2025-115), and the requirement for informed consent for this retrospective analysis was waived. All data were anonymized before analysis. The inclusion criteria were as follows: (1) pathologically confirmed advanced lung squamous cell carcinoma (AJCC 8th edition stage IIIB–IV) with measurable lesions; (2) failure of or intolerance to prior systemic therapy, with immunotherapy administered as first-line or subsequent systemic treatment; (3) hemoptysis requiring interventional management; (4) treatment with BACE with or without subsequent I-125 seed implantation; and (5) available clinical, imaging, and follow-up data for endpoint assessment. Exclusion criteria comprised: (1) active malignancies other than lung cancer that could independently affect survival assessment; (2) incomplete key clinical, procedural, or follow-up data; (3) uncontrolled coagulation disorders or severe cardiopulmonary, hepatic, renal, or neurologic dysfunction precluding interventional treatment; and (4) contraindications to BACE, including uncorrectable coagulopathy, severe contrast allergy, or inability to tolerate angiographic intervention. Patients were categorized according to the actual local treatment strategy received after BACE. Group G1 included patients treated with BACE plus immunotherapy; all immunotherapy was administered after BACE. Group G2 included patients who underwent I-125 seed implantation within 7 days after BACE and subsequently received immunotherapy. No patient in either group died on the day of BACE or within 7 days after BACE. The date of BACE was used as the baseline procedural date for outcome assessment. Baseline demographic and clinical characteristics are presented in Table 1 and Supplementary Tables S1 and S2.

### 2.2. Relationship with Previous Institutional Studies

Our institution has previously reported a related cohort evaluating 8Spheres microsphere embolization combined with I-125 seed implantation in patients with advanced LUSC complicated by hemoptysis. The present study differs from that previous report in enrollment period, treatment background, comparator framework, and clinical objective. Specifically, the current cohort included patients treated between June 2023 and June 2025 and focused on the incremental value of I-125 seed implantation in patients receiving BACE plus immunotherapy. Although there was a limited calendar overlap with the previous cohort between June 2023 and December 2023, anonymized patient identifiers, hospitalization numbers, treatment dates, and procedural records were cross-checked. Only 4 patients overlapped with the previous cohort, accounting for 4.4% of the present study population. Given the minimal overlap, all eligible patients were retained in the primary analysis.

### 2.3. Treatment Method

Before treatment, patients completed blood counts, urinalysis, coagulation testing, liver and renal function tests, infectious disease screening (hepatitis, syphilis, and HIV), electrocardiography, and contrast-enhanced chest CT. Written informed consent was obtained for BACE and related procedures. Patients in Group G2 also provided consent for I-125 seed implantation.

The BACE procedure began with a right femoral artery puncture. A 5F RLG or Cobra catheter (Terumo Co., Tokyo, Japan) was selectively advanced into the bronchial artery for angiography; supplementary angiography of the internal thoracic, diaphragmatic, and other relevant arteries was performed as needed to identify all tumor blood supply sources. Subsequently, a 2.7 F microcatheter (Hunan APT Medical Inc., Xiangxiang, Hunan, China) was used for superselective cannulation of the target vessel. Chemotherapy infusion was administered first using docetaxel (60 mg/m^2^) according to the institutional BACE protocol, with dose selection based on body surface area and patient tolerance [18]. Following chemotherapy, the target vessel was embolized with 300–500 μm 8Spheres microspheres (Jiangsu Hengrui Pharmaceuticals Co., Ltd., Lianyungang, Jiangsu, China) injected at a rate of 1 mL/min. Digital subtraction angiography (DSA) assessed embolization efficacy, with supplemental embolization performed if necessary.

Eligibility for I-125 seed implantation was determined by multidisciplinary review. The review considered tumor location, a safe puncture route, proximity to major vessels or mediastinal structures, coagulation status, pulmonary reserve, ECOG performance status, expected local control benefit, and patient preference. Patients who did not undergo implantation generally had no safe puncture route, excessive procedural risk, poor tolerance for an additional invasive procedure, refusal of implantation, or a clinical decision that BACE plus systemic therapy was the more appropriate option.

For I-125 seed implantation, performed 3–5 days post-BACE, the treatment plan was designed using a treatment planning system (TPS, Beijing Astro Technology Co. Ltd., Beijing, China), specifying the prescribed dose, needle insertion routes, and seed distribution. The seed sources (Beijing Atomic High-Tech Co., Ltd., Beijing, China) were 0.8 mm in diameter and 4.5 mm in length, with a fully enclosed titanium casing, an activity of 0.8 mCi, and a half-life of 59.6 days; the prescribed dose was 120–140 Gy. All implantations were performed under local anesthesia with CT guidance (Discovery RT, GE Healthcare; 5 mm slice thickness). An 18 G seed implantation needle (Hakko Co., Ltd., Chikuma-shi, Nagano, Japan) was used for puncture according to the TPS plan, with real-time contrast-enhanced scanning employed if tumor-adjacent major vessels were poorly demarcated. Seeds were implanted from deep to superficial layers at 0.5–1 cm intervals. CT was repeated immediately after implantation to check seed distribution and procedure-related complications. Postoperative D90 and V100 were used to assess dosimetric quality, and V150 and V200 were recorded when available. During follow-up, patients were evaluated for pneumothorax, pulmonary hemorrhage, infection, radiation pneumonitis, and seed migration.

Immunotherapy was administered at the treating oncologist’s discretion and according to patient tolerance. Tislelizumab was given intravenously at 200 mg every three weeks. In the G1 group, immunotherapy was generally initiated or resumed 3 days after BACE when the patient’s clinical condition was stable. In the G2 group, immunotherapy was generally initiated or resumed 3 days after I-125 seed implantation when clinically appropriate. No additional systemic therapy was administered during the analyzed treatment period, except for intra-arterial docetaxel delivered during BACE.

### 2.4. Outcome Definitions

The index date for all time-to-event analyses was the date of BACE. OS was defined as the time from BACE to death from any cause or last follow-up. PFS was defined as the time from BACE to radiologic disease progression or death from any cause. HFS was defined as the time from BACE to recurrent hemoptysis or last follow-up; patients without recurrent hemoptysis were censored at the last available follow-up. Immediate hemostatic response was assessed within 24 h after BACE. Complete resolution was defined as complete cessation of hemoptysis, and partial resolution was defined as a reduction of more than 70% in hemoptysis volume. Tumor response was evaluated according to RECIST version 1.1 using contrast-enhanced CT imaging. ORR was defined as complete response plus partial response, and DCR was defined as complete response, partial response, and stable disease. Adverse events were graded using CTCAE version 5.0 when the medical record contained sufficient detail.

### 2.5. Statistical Analysis

R software (version 4.4.1) was used for all analyses. Continuous variables are reported as mean ± standard deviation when normally distributed and as median (interquartile range) otherwise. Between-group comparisons used independent-samples t-tests. Categorical variables are reported as number (%) and were compared with chi-square or Fisher’s exact tests.

OS, PFS, and HFS were estimated with Kaplan–Meier curves and compared between groups with the log-rank test. Cox regression was used to calculate HRs and 95% confidence intervals. Because treatment allocation was not randomized, baseline imbalance was addressed with two approaches. First, propensity score matching was performed with the MatchIt package in R using a 1:1 ratio, a caliper of 0.1, and the following covariates: age, ECOG performance status, tumor size, TNM stage, metastasis status, hemoptysis severity, and immunotherapy exposure. Second, IPTW was used as the primary adjustment method to balance baseline covariates between groups. In the weighted cohort, continuous variables were compared with weighted t-tests, categorical variables with Rao and Scott adjusted chi-square tests, survival curves with weighted log-rank tests, and HRs with weighted Cox regression. Covariate balance was assessed using the standardized mean difference (SMD), with SMD < 0.1 considered acceptable. These methods reduce measured baseline imbalance but do not remove the possibility of unmeasured confounding.

Subgroup analyses were displayed as forest plots for clinically relevant strata. Treatment-by-subgroup interaction was tested with likelihood ratio tests.

All tests were two-sided, with *p* < 0.05 considered statistically significant.

## 3. Results

### 3.1. Baseline Characteristics

**Figure 1:**
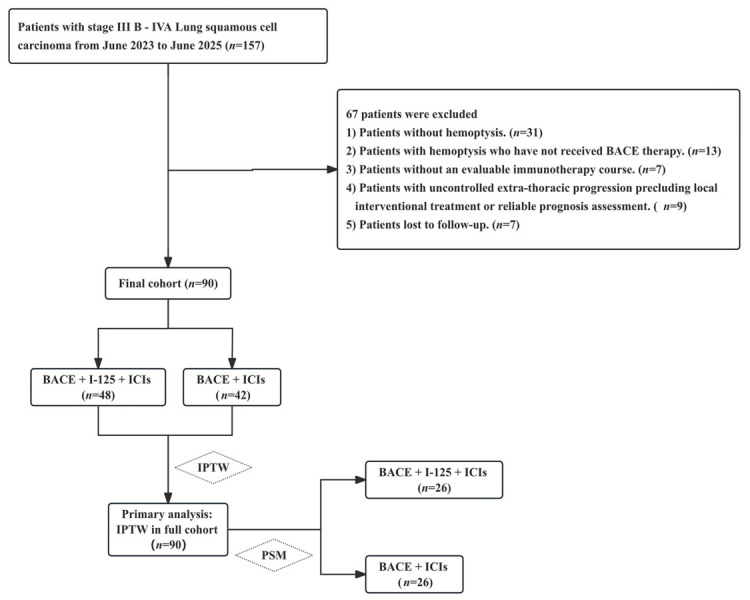
The study cohort screening process.

**Figure 2:**
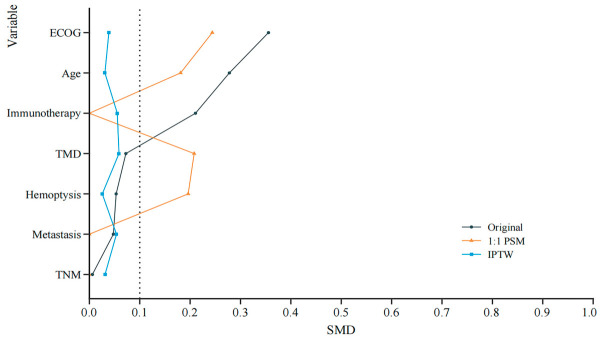
Covariate balance before and after IPTW adjustment. Standardized mean differences below 0.1 were considered indicative of acceptable balance. The vertical dashed line indicates the prespecified balance threshold of SMD = 0.1; values to the left of this line were considered indicative of acceptable covariate balance.

A total of 269 patients with advanced refractory lung squamous cell carcinoma were screened, from which 90 patients meeting the inclusion and exclusion criteria and receiving BACE plus immunotherapy were enrolled (Figure 1), including 42 in Group G1 and 48 in Group G2. To mitigate measurable confounding, inverse probability of treatment weighting (IPTW) was used to construct a weighted cohort for the primary analysis. IPTW adjustment improved covariate balance between groups, as shown in Table 1 and Table S1. A 1:1 propensity score matching (PSM) was also performed as a sensitivity analysis. Following PSM, each group contained 26 patients, achieving acceptable covariate balance (Table S2), with standardized mean differences (SMD) below 0.1 for most variables (Figure 2). In the IPTW-weighted cohort, the median age was 67.00 years (61.00–69.00) in G1 and 67.00 years (63.00–69.00) in G2, with 38 (88.4%) and 39 (83.0%) males, respectively. For Group G2, the mean number of implanted particles was 48.23 ± 17.18, the median postoperative verified D90 dose was 131.89 Gy (126.15–134.60), and the mean V100 percentage was 96.66 ± 1.04% (Table S1). No significant differences were observed between groups in age, gender, smoking history, comorbidities, hemoptysis volume, maximum tumor diameter, or TNM stage (all *p* > 0.05). Detailed clinical characteristics are summarized in Table 1.

### 3.2. Clinical Outcomes

**Figure 3:**
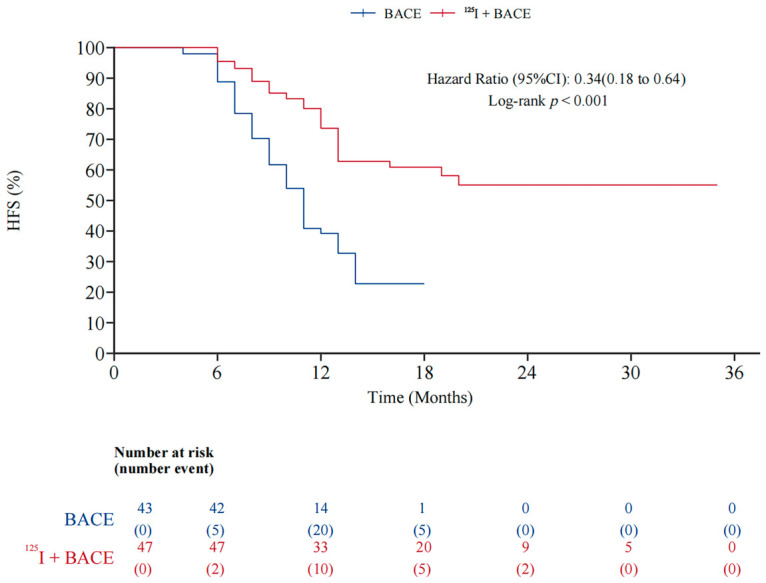
IPTW-adjusted hemoptysis-free survival curves comparing BACE plus. immunotherapy with BACE, I-125 seed implantation, and immunotherapy.

**Figure 4:**
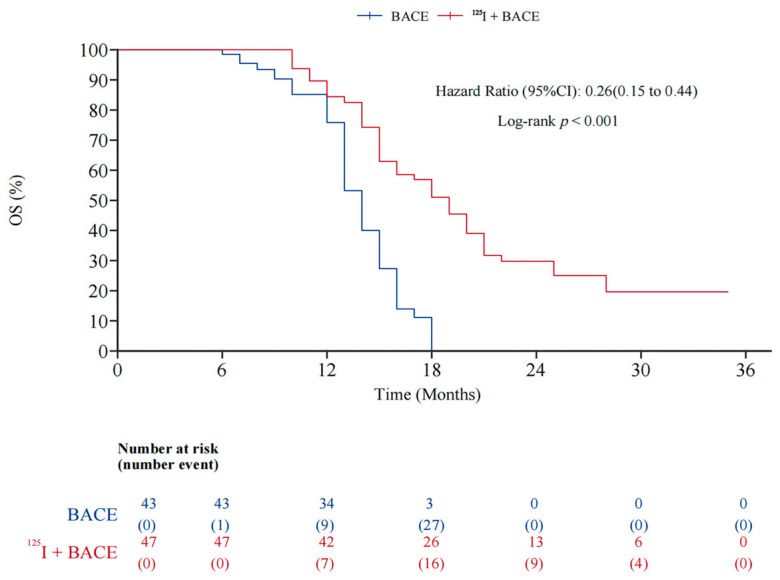
IPTW-adjusted overall survival curves comparing BACE plus immunotherapy with BACE, I-125 seed implantation, and immunotherapy.

In the IPTW-weighted cohort, the G2 group demonstrated superior hemoptysis-free survival (HFS) compared to the G1 group (Figure 3). The median HFS was not reached versus 11 months (HR = 0.34, 95% CI 0.18–0.64, *p* < 0.05). The 1-year HFS rate was 73.6% (95% CI, 61.5–88.1%) in G2 versus 39.2% (95% CI, 25.7–59.8%) in G1 (Table 2), indicating an added benefit of I-125 seeds in achieving sustained hemostasis. Subsequent analysis of overall survival (OS) also favored the G2 group (Figure 4). The median OS was 19 versus 14 months (HR = 0.26, 95% CI 0.15–0.44, *p* < 0.05), with 1-year OS rates of 84.4% (95% CI, 74.9–95.2%) in G2 and 75.9% (95% CI, 63.8–90.2%) in G1 (Table 2). The HFS and OS outcomes in the PSM-matched cohort were consistent with the primary analysis (Figures S1 and S2), suggesting that the observed associations were generally consistent across different adjustment methods.

**Figure 5:**
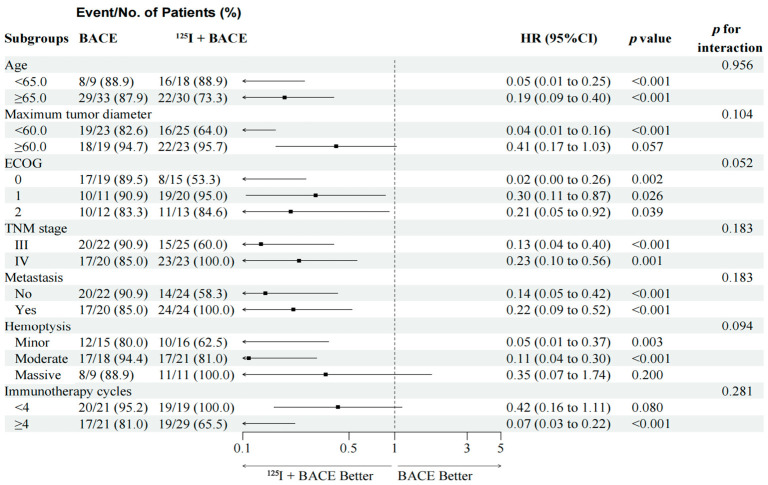
Subgroup analysis of overall survival. Hazard ratios less than 1 favor the I-125 seed implantation group. Interaction *p* values were calculated to assess heterogeneity of treatment effect across subgroups. Squares indicate the hazard ratio point estimates, and horizontal lines indicate the corresponding 95% confidence intervals. Arrows indicate that the confidence interval extends beyond the displayed x-axis range. The vertical dashed line represents HR = 1, indicating no difference between treatment groups.

The rates of complete resolution (CR, complete cessation of hemoptysis) and partial resolution (PR, >70% reduction in hemoptysis) within 24 h post-procedure were 72.3% versus 67.4% and 27.7% versus 32.6%, respectively (*p* > 0.05). At 6 months postoperatively, G2 showed higher objective response rate (ORR) and disease control rate (DCR) than G1 (ORR: 85.1% vs. 62.8%; DCR: 93.6% vs. 79.1%; *p* < 0.05), along with a longer median progression-free survival (PFS) (12.0 vs. 9.0 months, HR = 0.34, 95% CI 0.21–0.56, *p* < 0.05) (Table 2). These findings suggest that adding I-125 seed implantation improved tumor response and prolonged PFS in this selected cohort. Regarding safety, the most common adverse events included nausea, fever, chest pain, and pneumothorax (Table 2). Pneumothorax occurred in five patients in G2 and resolved with conservative or symptomatic management. Interstitial pneumonia potentially related to immunotherapy was recorded in 3 patients in Group G1 and 6 patients in Group G2. No grade 3 or higher adverse events or other immunotherapy-related adverse events were noted in the available medical records. No treatment-related deaths were documented. These findings support acceptable short-term safety in the available records, but longer-term prospective safety monitoring is needed. Pre-specified subgroup analyses demonstrated consistent directional benefits of the combination strategy on OS across major subgroups (Figure 5). Interaction tests revealed no significant heterogeneity (*p* for interaction >0.05 for all), indicating a stable treatment effect. These analyses were exploratory because of the limited sample size.

## 4. Discussion

In this retrospective IPTW-adjusted cohort of patients with advanced refractory lung squamous cell carcinoma (LUSC) complicated by hemoptysis, the integration of I-125 seed implantation with bronchial artery chemoembolization (BACE) and immunotherapy was associated with longer overall survival (OS), hemoptysis-free survival (HFS), and progression-free survival (PFS), along with improved 6-month tumor response (median OS: 19 vs. 14 months; median HFS: not reached vs. 11 months; median PFS: 12 vs. 9 months; *p* < 0.05). These findings imply that I-125 seed implantation may confer additional local control benefit in selected patients undergoing BACE and immunotherapy. The continuous release of gamma rays from I-125 seeds inhibits tumor growth and blocks neoangiogenesis, exerting a synergistic effect with BACE. This demonstrates the potential advantage of this regimen, offering a safe and effective therapeutic strategy for advanced LUSC with hemoptysis.

For patients with advanced refractory LUSC, hemoptysis represents both an acute emergency and a potential cause for interrupting subsequent treatments [19]. Our findings suggest that combined therapy provides more durable local control, transforming recurrent hemoptysis into a stably managed condition. This stability enables patients to complete subsequent courses of immunotherapy more effectively. The value of this approach thus extends beyond symptom control to optimizing the overall treatment trajectory. Patients with LUSC characterized by a high tumor burden and rich vascular supply face greater risks of local progression and massive hemoptysis [20,21,22]. For such patients, an initial intensive local strategy involving I-125 seed implantation—shifting from reactive treatment to proactive prevention—may represent a more rational approach.

I-125 seed implantation is an established brachytherapy technique. Studies by Aljabab et al. [23] indicate that radiotherapy provides acceptable short-term efficacy for lung cancer-related hemoptysis. Compared to external beam radiotherapy (EBRT) or stereotactic body radiotherapy (SBRT), brachytherapy allows precise targeting and is widely used in treating solid tumors such as prostate, liver, and pancreatic cancers [14,16,24,25]. With an effective radius of only 1.7 cm, it destroys tumor cells while sparing surrounding healthy tissue. Furthermore, the seeds have an effective half-life of 59.6 days, continuously releasing low-energy gamma rays within the target area. This causes cumulative damage to tumor cells in the G2 and M phases, ultimately inducing apoptosis [26]. By controlling the local tumor, this approach also slows its invasion into adjacent major vascular structures like the bronchial and pulmonary arteries [27]. I-125 seeds therefore effectively reduce the risk of recurrent hemoptysis through local tumor control, contributing to superior hemoptysis-free survival.

The role of brachytherapy in lung cancer has also been investigated in randomized trials. In ACOSOG Z4032 [28], adding brachytherapy to sublobar resection did not clearly improve local control in high-risk patients with early-stage non-small cell lung cancer, indicating that its benefit may depend on the clinical context. Unlike that study, our study included patients with advanced refractory LUSC complicated by hemoptysis, and I-125 seeds were implanted after BACE to deliver sustained local irradiation for tumor-related bleeding control. Accordingly, our findings suggest a possible benefit in this specific palliative interventional setting.

BACE achieves immediate hemostasis by embolizing tumor-feeding arteries. The local perfusion of high-concentration chemotherapeutic agents simultaneously delivers greater cytotoxic effects to the tumor while reducing systemic toxicity. In the study by Liu et al. [29], the BACE group showed superior PFS and OS compared to systemic chemotherapy, with fewer adverse events (all *p* < 0.05). However, residual tumor cells and the establishment of collateral circulation after BACE can lead to local tumor progression and recurrent hemoptysis [30]. Following BACE, I-125 seeds provide nearly six months of sustained brachytherapy. This sequential approach synergistically complements BACE, jointly controlling local tumor progression. The dual strategy addresses hemoptysis while inhibiting tumor growth and angiogenesis. In Tang et al.’s study [31], BACE combined with I-125 seeds significantly improved OS (27.5 vs. 15 months, *p* < 0.001) in 45 patients with non-small cell lung cancer who had failed standard therapy. Moreover, the local hypoxic environment induced by BACE may suppress the proliferation of residual tumor cells, potentially increasing their sensitivity to subsequent radiotherapy and enhancing the efficacy of I-125 seeds.

Local therapy must be integrated with systemic treatment to achieve maximal efficacy. Immunotherapy has significantly improved outcomes for advanced, refractory squamous cell lung cancer in recent years. Theelen et al. [32] demonstrated that combining pembrolizumab with radiotherapy yielded a significantly longer median OS than immunotherapy alone (19.2 vs. 8.7 months), suggesting radiotherapy may enhance the systemic antitumor immune response. Zhou et al. [33] also found that sugemalimab combined with radiotherapy significantly prolonged median PFS (9.0 vs. 5.8 months). Radiotherapy not only exerts local cytotoxic effects but can also promote a systemic immune response by inducing immunogenic cell death, releasing tumor-associated antigens, and activating the cGAS–STING/type I interferon axis [34]. This process facilitates dendritic cell cross-presentation and T-cell activation. Radiotherapy may also increase PD-L1 expression in the tumor microenvironment, which supports combining radiation with immune checkpoint blockade [35]. ICIs may further normalize tumor vasculature through T cell-dependent mechanisms, improving perfusion and reducing hypoxia. In this context, I-125 seed brachytherapy may provide sustained low-dose-rate irradiation, which may alter the local immune microenvironment [36]. Such effects could increase antigen release and innate immune activation, although distant effects remain uncertain. Sui et al. [37] reported clinical improvement in three patients who received immunotherapy after I-125 seed implantation. Our findings extend this observation to advanced refractory LUSC complicated by hemoptysis.

In this cohort, treatment was organized around three targets: embolization for tumor-feeding vessels, brachytherapy for local tumor control, and immunotherapy for systemic disease. This sequence reflects a practical local-systemic strategy. In selected patients with advanced malignancy, local therapy should not be limited to palliation when it can help stabilize disease and allow systemic treatment to continue.

In China, CT-guided I-125 seed implantation has been adopted in many tertiary general hospitals with interventional oncology and brachytherapy capabilities. However, its safety and reproducibility depend on operator experience, standardized treatment planning, precise recognition of the target lesion and adjacent critical structures, and the ability to manage complications such as pneumothorax, bleeding, and seed migration. Therefore, broader use of this strategy requires careful patient selection, multidisciplinary support, procedural standardization, and structured operator training.

For carefully selected patients with advanced refractory LUSC and hemoptysis who have adequate performance status and a technically feasible puncture route, integrating I-125 seed implantation with BACE and immunotherapy may represent a viable local-systemic treatment strategy. For those at high puncture-related risk or with poor procedural tolerance, BACE combined with immunotherapy remains a reasonable option.

This study has several limitations. First, this was a retrospective single-center study with non-randomized treatment allocation. Although IPTW and PSM were used to reduce measured baseline imbalances, treatment selection was influenced by technical feasibility, physician judgment, and patient preference; therefore, residual confounding from unmeasured factors cannot be excluded. Second, the sample size was relatively small, particularly for subgroup analyses, which should be considered exploratory. Third, long-term safety data after I-125 seed implantation were limited, and immune-related adverse events were assessed retrospectively from available medical records; therefore, low-grade events may have been underreported. In addition, standardized quality-of-life measures, PD-L1 status, previous thoracic radiotherapy exposure, and detailed molecular data were incompletely documented in some patients, preventing reliable adjustment for these potentially important factors. Finally, although CT-guided I-125 seed implantation has been adopted in many hospitals in China, its safety and reproducibility may still depend on operator experience, puncture skills, accurate recognition of adjacent critical structures, and the ability to manage procedure-related complications. Prospective multicenter studies with larger sample sizes, standardized toxicity monitoring, patient-reported outcome assessment, and biomarker evaluation are warranted.

## 5. Conclusions

In this retrospective IPTW-adjusted cohort, adding I-125 seed implantation to BACE plus immunotherapy was associated with longer HFS, improved tumor response, and extended PFS and OS in selected patients. However given this study employed a non-randomized design, had a limited sample size, and lacked complete data on biomarkers, quality of life, and long-term safety, the findings need to be confirmed through a prospective, multicenter validation study.

## Figures and Tables

**Table 1:** Baseline characteristics (IPTW).

Variables	Total (Weighted *n* = 90)	BACE (Weighted *n* = 43)	^125^I + BACE (Weighted *n* = 47)	*p*
Sex, *n* (%)				0.351
-Male	77 (85)	38 (89)	39 (83)	
-Female	13 (15)	5 (11)	8 (17)	
Age, M (Q1, Q3)	67.00 (62.00, 69.00)	67.00 (61.00, 69.00)	67.00 (63.00, 69.00)	0.842
Smoking history, weighted *n* (%)				0.925
-No	32 (36)	16 (37)	17 (36)	
-Yes	57 (64)	27 (63)	30 (64)	
No. of Co-morbidity, weighted *n* (%)				0.410
1	51 (57)	27 (64)	24 (52)	
2	13 (15)	4 (10)	9 (19)	
3	25 (28)	11 (26)	14 (29)	
ECOG PS, weighted *n* (%)				0.985
0	32 (36)	15 (36)	17 (36)	
1	32 (36)	16 (37)	17 (35)	
2	25 (28)	12 (27)	14 (29)	
Tumor location, weighted *n* (%)				0.200
-RML	11 (13)	7 (16)	5 (10)	
-RUL	13 (14)	4 (9.9)	8 (18)	
-RLL	36 (40)	19 (44)	17 (36)	
-LUL	22 (24)	12 (28)	10 (21)	
-LLL	8 (8.8)	1 (2.0)	7 (15)	
Maximum tumor diameter (mm), M (Q1, Q3)	57.00 (45.00, 67.00)	57.00 (45.00, 67.00)	57.00 (38.00, 67.00)	0.942
TNM stage, weighted *n* (%)				0.890
-III	48 (54)	23 (54)	25 (53)	
-IV	42 (46)	19 (46)	22 (47)	
Metastasis, weighted *n* (%)				0.890
-No	48 (54)	23 (54)	25 (53)	
-Yes	42 (46)	19 (46)	22 (47)	
Hemoptysis, weighted *n* (%)				0.994
-Minor (<100 mL/24 h)	33 (37)	16 (37)	18 (38)	
-Moderate (100–500 mL/24 h)	37 (41)	18 (41)	19 (41)	
-Massive (>500 mL/24 h or >100 mL per time)	19 (22)	9 (22)	10 (21)	
Hemoglobin drop (g/L), M (Q1, Q3)	12.00 (7.00, 15.00)	12.00 (7.00, 16.00)	12.00 (6.00, 15.00)	0.550
Previous hemostatic treatment, weighted *n* (%)				0.393
-No	49 (55)	26 (60)	24 (50)	
-Tranexamic acid (intravenous)	40 (45)	17 (40)	23 (50)	
Immunotherapy cycles, weighted *n* (%)				0.805
<4	38 (42)	19 (44)	19 (41)	
≥4	52 (58)	24 (56)	28 (59)	

Abbreviations: SD: standard deviation, M: Median, Q1: 1st Quartile, Q3: 3rd Quartile, RUL: Right Upper Lobe, RML: Right Middle Lobe, RLL: Right Lower Lobe, LUL: Left Upper Lobe, LLL: Left Lower Lobe; ECOG PS, Eastern Cooperative Oncology Group Performance Status. Notes: Continuous data presented as M (Q1–Q3) (non-normally distributed). Categorical data presented as weighted *n* (%). Because IPTW produces fractional weighted counts, displayed weighted counts and percentages were rounded; therefore, categorical sums may differ slightly from the displayed weighted column totals.

**Table 2:** Clinical outcomes and safety of BACE + ^125^I + ICIs vs BACE + ICIs.

Outcome	BACE (*n* = 43)	^125^I + BACE (*n* = 47)	HR (95% CI)	*p*
Follow-up and event counts				
Death events, *n* (%)	88.3% (38/43)	82.9% (39/47)		0.467
HFS events (re-hemoptysis), *n* (%)	67.4% (29/43)	40.4% (19/47)		0.010
Survival endpoints				
Median OS, months (95% CI)	14 (13.0–15.0)	19 (16.0–21.0)	0.26 (0.15–0.44)	<0.001
1 year OS rate, % (95% CI)	75.9% (63.8–90.2)	84.4% (74.9–95.2)		0.276
Median HFS, months (95% CI)	11.0 (9.0–14.0)	NR (13.0–NR)	0.34 (0.18–0.64)	<0.001
1 year HFS rate, % (95% CI)	39.2 (25.7–59.8)	73.6 (61.5–88.1)		0.002
Median PFS, months (95% CI)	9.0 (8. 0–11.0)	12.0 (11.0–16.0)	0.34 (0.21–0.56)	<0.001
1 year PFS rate, % (95% CI)	15.9 (7.3–34.8)	47.7 (35.1–64.7)		<0.001
Efficacy Comparison				
24 h hemostasis				0.073
-CR, *n* (%)	67.4% (29/43)	72.3% (34/47)		
-PR, *n* (%)	32.6% (14/43)	27.7% (13/47)		
ORR(CR+PR), *n* (%)	62.7% (27/43)	85.1% (40/47)		0.015
DCR (CR+PR+SD), *n* (%)	79.1% (34/43)	93.6% (44/47)		0.043
Adverse events				
Pneumothorax	0	10.6% (5/47)		-
Chest Pain	16.3% (7/43)	21.3% (10/47)		0.545
Fever	18.6% (8/43)	12.8% (6/47)		0.445
Nausea	18.6% (8/43)	23.4% (11/47)		0.577
Interstitial pneumonia	7.0% (3/43)	12.8% (6/47)		0.489

Abbreviations: BACE, bronchial artery chemoembolization; ICI, immune checkpoint inhibitor; OS, overall survival; HFS, hemoptysis-free survival; PFS, progression-free survival; CR, complete response; PR, partial response; SD, stable disease; ORR, objective response rate; DCR, disease control rate; HR, hazard ratio; CI, confidence interval; NR, not reached. OS, HFS and PFS were analyzed in the IPTW-weighted cohort using weighted Cox models and log-rank tests; hemostasis and complications were compared using χ^2^ tests. Data are presented as number (%) unless otherwise specified.

## Data Availability

The original contributions presented in this study are included in the article/Supplementary Material. Further inquiries can be directed to the corresponding authors.

## Supplementary Materials

The following supporting information can be downloaded at: https://www.mdpi.com/article/10.3390/curroncol33070402/s1, Figure S1: Hemoptysis-free survival after propensity score matching; Figure S2: Overall survival after propensity score matching; Table S1: Baseline characteristics; Table S2: Baseline characteristics after propensity score matching.

## Abbreviations

The following abbreviations are used in this manuscript:

BACE	bronchial artery infusion chemotherapy embolization
CI	confidence interval
CR	complete response
DCR	disease control rate
DSA	Digital subtraction angiography
EBRT	external beam radiotherapy
HFS	hemoptysis-free survival
HR	hazard ratios
ICIs	Immune checkpoint inhibitors
I-125	Iodine-125
IPTW	inverse probability of treatment weighting
LUSC	lung squamous cell carcinoma
ORR	objective response rate
OS	overall survival
PFS	progression-free survival
PR	partial response
PSM	propensity score matching
SBRT	stereotactic body radiotherapy
SMD	standardized mean difference
TPS	treatment planning system
