# Supplementary material for: Incremental Value of Iodine-125 Seed Implantation After Bronchial Artery Chemoembolization in Immunotherapy-Treated Advanced Lung Squamous Cell Carcinoma with Hemoptysis: A Retrospective Cohort Study Using Inverse Probability of Treatment Weighting
Source: Curr Oncol. 2026 Jul 5;33(7):402. doi: 10.3390/curroncol33070402 (PMC13409501; doi:10.3390/curroncol33070402)
Supplement: Supplementary file 1 [file curroncol-33-00402-s001.zip › Table S1.pdf]

Table S1. Baseline characteristics

Variables	Total (N = 90)	BACE (N = 42)	^{125}I + BACE (N = 48)	P
Sex， n (%)				0.320
-Male	78 (86.7)	38 (90.5)	40 (83.3)	
-Female	12 (13.3)	4 (9.5)	8 (16.7)	
Age, M (Q1, Q3)	67.00 (62.25, 69.00)	68.00 (65.00, 69.75)	66.00 (61.50, 68.25)	0.105
Smoking history, n(%)				0.638
-No	32 (35.6)	16 (38.1)	16 (33.3)	
-Yes	58 (64.4)	26 (61.9)	32 (66.7)	
No. of Co-morbidity, n(%)				0.577
1	49 (54.4)	25 (59.5)	24 (50)	
2	14 (15.6)	5 (11.9)	9 (18.8)	
3	27 (30)	12 (28.6)	15 (31.2)	
ECOG PS, n(%)				0.255
0	34 (37.8)	19 (45.2)	15 (31.2)	
1	31 (34.4)	11 (26.2)	20 (41.7)	
2	25 (27.8)	12 (28.6)	13 (27.1)	
Tumor location, n(%)				0.195
-RML	8 (8.9)	4 (9.5)	4 (8.3)	
-RUL	13 (14.4)	5 (11.9)	8 (16.7)	
-RLL	37 (41.1)	20 (47.6)	17 (35.4)	
-LUL	23 (25.6)	12 (28.6)	11 (22.9)	
-LLL	9 (10)	1 (2.4)	8 (16.7)	
Maximum tumor diameter (mm), M (Q1, Q3)	57.00 (45.00, 67.00)	55.00 (45.00, 67.00)	57.50 (44.75, 67.00)	0.668
TNM stage, n(%)				0.978
-III	47 (52.2)	22 (52.4)	25 (52.1)	
-IV	43 (47.8)	20 (47.6)	23 (47.9)	
Metastasis, n (%)				0.978
-No	47 (52.2)	22 (52.4)	25 (52.1)	
-Yes	43 (47.8)	20 (47.6)	23 (47.9)	
Hemoptysis, n(%)				0.969
-Minor (<100 mL/24h)	31 (34.4)	15 (35.7)	16 (33.3)	
-Moderate (100 – 500 mL/24h)	39 (43.3)	18 (42.9)	21 (43.8)	
-Massive (>500 mL/24h or >100mL per time)	20 (22.2)	9 (21.4)	11 (22.9)	
Hemoglobin drop (g/L), M (Q1, Q3)	12.00 (7.00, 15.75)	12.50 (7.25, 15.75)	12.00 (7.00, 15.25)	0.519
Previous hemostatic treatment, n(%)				0.271
-No	48 (53.3)	25 (59.5)	23 (47.9)	
-Tranexamic acid	42 (46.7)	17 (40.5)	25 (52.1)	

Variables	Total (N = 90)	BACE (N = 42)	^{125}I + BACE (N = 48)	P
(intravenous)				
Immunotherapy cycles, n (%)				0.321
<4	40 (44.4)	21 (50)	19 (39.6)	
≥4	50 (55.6)	21 (50)	29 (60.4)	
No. of seeds, Mean ± SD			48.23 ± 17.18	
D90 (Gy), M (Q1, Q3)			131.89 (126.15,134.60)	
V100 (%), Mean ± SD			96.66 ± 1.04	

Abbreviations: SD: standard deviation, M: Median, Q1: 1st Quartile, Q3: 3rd Quartile, RUL: Right Upper Lobe, RML: Right Middle Lobe, RLL: Right Lower Lobe, LUL: Left Upper Lobe, LLL: Left Lower Lobe; ECOG PS, Eastern Cooperative Oncology Group Performance Status.

Notes:

Continuous data presented as Mean ± SD (normally distributed) or M (Q1, Q3) (non-normally distributed).
Categorical data presented as n (%).
