# Supplementary material for: Incremental Value of Iodine-125 Seed Implantation After Bronchial Artery Chemoembolization in Immunotherapy-Treated Advanced Lung Squamous Cell Carcinoma with Hemoptysis: A Retrospective Cohort Study Using Inverse Probability of Treatment Weighting
Source: Curr Oncol. 2026 Jul 5;33(7):402. doi: 10.3390/curroncol33070402 (PMC13409501; doi:10.3390/curroncol33070402)
Supplement: Supplementary file 1 [file curroncol-33-00402-s001.zip › Table S2.pdf]

Table S2. Baseline characteristics after propensity score matching

Variables	Total (N = 52)	BACE (N = 26)	^{125}I + BACE (N = 26)	P
Sex， n (%)				1.000
-Male	44 (84.6)	22 (84.6)	22 (84.6)	
-Female	8 (15.4)	4 (15.4)	4 (15.4)	
Age, M (Q1, Q3)	67.00 (62.75, 69.25)	68.00 (65.00, 69.75)	66.50 (62.25, 68.75)	0.388
Smoking history, n(%)				0.569
-No	20 (38.5)	11 (42.3)	9 (34.6)	
-Yes	32 (61.5)	15 (57.7)	17 (65.4)	
No. of Co-morbidity, n(%)				0.919
1	26 (50)	13 (50)	13 (50)	
2	9 (17.3)	4 (15.4)	5 (19.2)	
3	17 (32.7)	9 (34.6)	8 (30.8)	
ECOG PS, n(%)				0.683
0	19 (36.5)	8 (30.8)	11 (42.3)	
1	15 (28.8)	8 (30.8)	7 (26.9)	
2	18 (34.6)	10 (38.5)	8 (30.8)	
Tumor location, n(%)				0.289
-RML	6 (11.5)	3 (11.5)	3 (11.5)	
-RUL	5 (9.6)	2 (7.7)	3 (11.5)	
-RLL	22 (42.3)	12 (46.2)	10 (38.5)	
-LUL	15 (28.8)	9 (34.6)	6 (23.1)	
-LLL	4 (7.7)	0 (0)	4 (15.4)	
Maximum tumor diameter (mm), M (Q1, Q3)	53.00 (44.00, 65.50)	52.00 (44.25, 61.75)	57.00 (44.25, 67.00)	0.379
TNM stage, n(%)				1.000
-III	28 (53.8)	14 (53.8)	14 (53.8)	
-IV	24 (46.2)	12 (46.2)	12 (46.2)	
Metastasis, n (%)				1.000
-No	28 (53.8)	14 (53.8)	14 (53.8)	
-Yes	24 (46.2)	12 (46.2)	12 (46.2)	
Hemoptysis, n(%)				0.781
-Minor (<100 mL/24h)	21 (40.4)	10 (38.5)	11 (42.3)	
-Moderate (100－500 mL/24h)	21 (40.4)	10 (38.5)	11 (42.3)	
-Massive (>500 mL/24h or >100mL per time)	10 (19.2)	6 (23.1)	4 (15.4)	
Hemoglobin drop (g/L), M (Q1, Q3)	12.00 (6.00, 15.25)	13.00 (9.00, 15.75)	10.00 (6.00, 14.75)	0.226
Previous hemostatic treatment, n(%)				0.575
-No	30 (57.7)	16 (61.5)	14 (53.8)	
-Tranexamic acid	22 (42.3)	10 (38.5)	12 (46.2)	

Variables	Total (N = 52)	BACE (N = 26)	^{125}I + BACE (N = 26)	P
(intravenous)				
Immunotherapy cycles, n (%)				1.000
<4	20 (38.5)	10 (38.5)	10 (38.5)	
≥4	32 (61.5)	16 (61.5)	16 (61.5)	

Abbreviations: SD: standard deviation, M: Median, Q1: 1st Quartile, Q3: 3rd Quartile, RUL: Right Upper Lobe, RML: Right Middle Lobe, RLL: Right Lower Lobe, LUL: Left Upper Lobe, LLL: Left Lower Lobe; ECOG PS, Eastern Cooperative Oncology Group Performance Status.

Notes:

Continuous data presented as Mean ± SD (normally distributed) or M (Q1, Q3) (non-normally distributed).

Categorical data presented as n (%).
