# Supplementary figures and images for: Incremental Value of Iodine-125 Seed Implantation After Bronchial Artery Chemoembolization in Immunotherapy-Treated Advanced Lung Squamous Cell Carcinoma with Hemoptysis: A Retrospective Cohort Study Using Inverse Probability of Treatment Weighting
Source: Curr Oncol. 2026 Jul 5;33(7):402. doi: 10.3390/curroncol33070402 (PMC13409501; doi:10.3390/curroncol33070402)

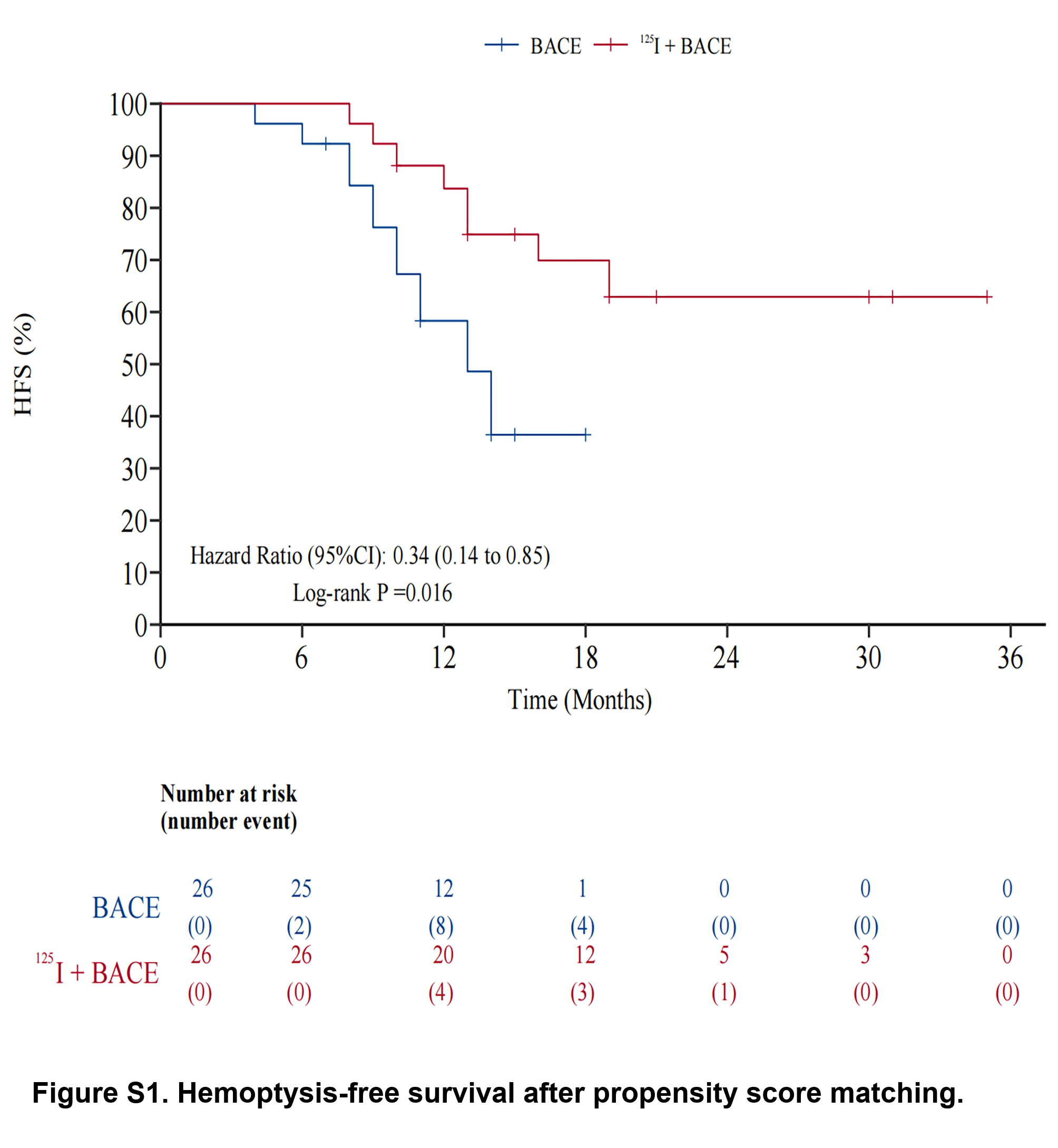

Supplement: Supplementary file 1 [file curroncol-33-00402-s001.zip › Figure S1.tif]

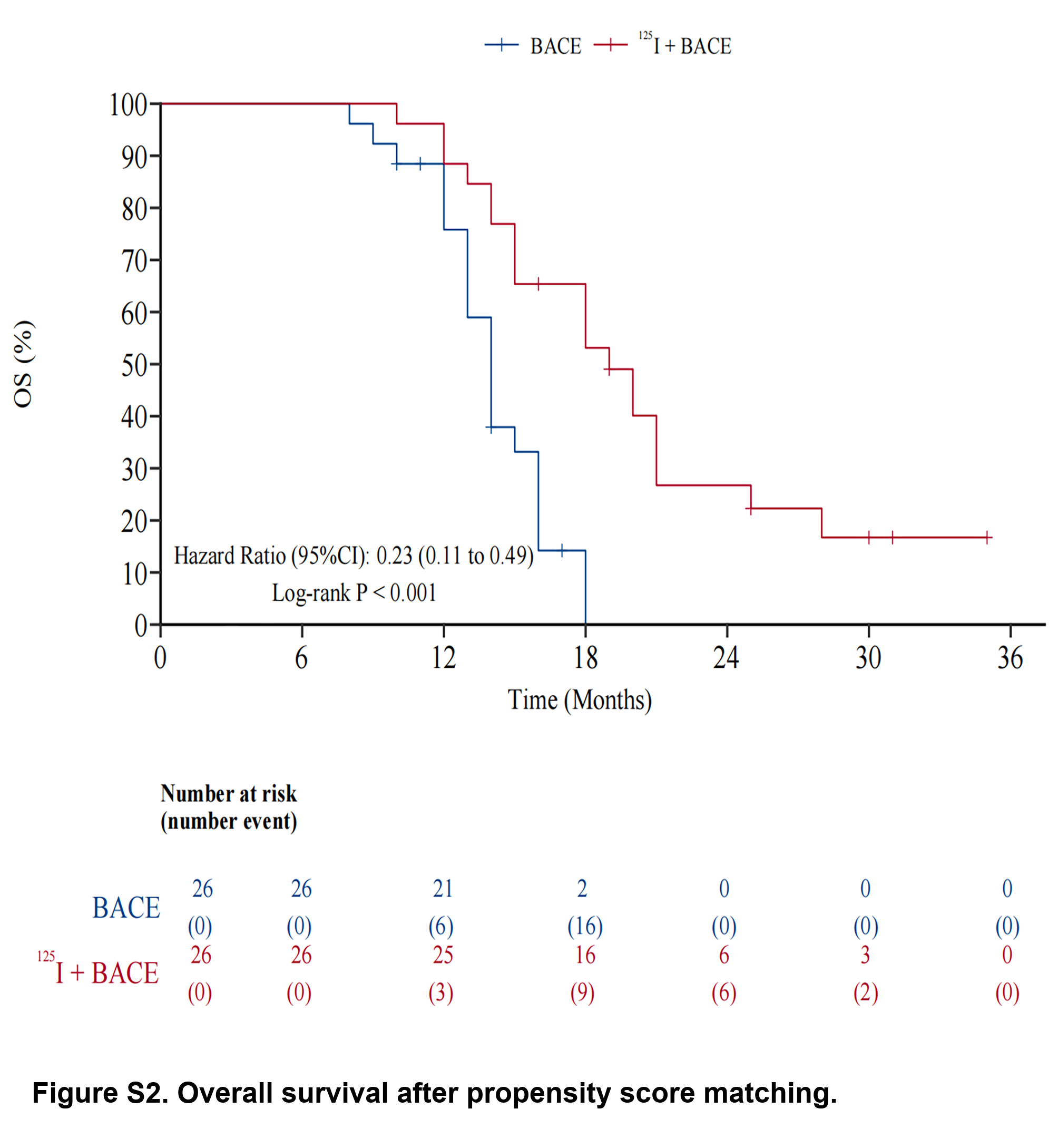

Supplement: Supplementary file 1 [file curroncol-33-00402-s001.zip › Figure S2.tif]
